# Unveiling the Possible Role of Neuroprotective Efficacy of Hordenine (HOR) Barley Derived Natural Product Against Scopolamine Induced Alzheimer's Disease & Neurotoxicity in Experimental Rats

**DOI:** 10.1002/alz70859_098761

**Published:** 2025-12-25

**Authors:** Manmohan Singhal, Mohit Agrawal

**Affiliations:** ^1^ Faculty of Pharmacy, School of Pharmaceutical & Population Health Informatics, DIT University, Dehradun, Uttrakhand India; ^2^ School of Medical & Allied Sciences, K.R. Mangalam University, Gurugram, Haryana India

## Abstract

**Background:**

Alzheimer's disease (AD), the leading etiology of progressive dementia in the aging population, is a chronic neurodegenerative disorder characterized by a gradual decline in cognitive functions. Despite the unclear etiology, two major pathogenic mechanisms have been identified: a deficiency in brain acetylcholine levels and the presence of oxidative stress. The aim of the present study was to evaluate the potential anti‐Alzheimer effects of hordenine in mitigating scopolamine‐induced behavioral and neurochemical alterations in rats.

**Method:**

Hordenine (50 and 100 mg/kg) and donepezil (5 mg/kg) were administered orally for seven consecutive days. At the conclusion of the treatment period, dementia was induced by a single intraperitoneal injection of scopolamine (20 mg/kg). Behavioral assessments, including the conditioned avoidance and Y‐maze tests, were performed 30 minutes post‐injection. Subsequently, the rats were euthanized, and brain homogenates were collected for the measurement of neurotransmitter levels (noradrenaline, dopamine, serotonin, and γ‐aminobutyric acid) as well as acetylcholinesterase activity. In addition, inflammatory and oxidative stress markers were evaluated, and histopathological examinations were conducted to further investigate the effects of the treatments.

**Result:**

Scopolamine‐treated rats exhibited significant alterations in neurobehavioral patterns and cognitive deficits, consistent with the induction of experimental dementia. Specifically, scopolamine administration led to an increase in midbrain acetylcholinesterase (AChE) activity, as well as elevated levels of lipid peroxidation (LPO), nitrite, tumor necrosis factor‐alpha (TNF‐α), interleukin‐1 beta (IL‐1β), nuclear factor‐kappa B (NF‐κB), and brain‐derived neurotrophic factor (BDNF). However, treatment with donepezil and hordenine effectively ameliorated these neurochemical and behavioral changes, restoring normal levels of the aforementioned markers and demonstrating a robust neuroprotective effect against scopolamine‐induced Alzheimer's‐like pathology. These findings suggest that both donepezil and hordenine may offer potential therapeutic benefits in mitigating the pathophysiological features of Alzheimer's disease.

**Conclusion:**

Hordenine administration resulted in significant improvements in memory and cognitive function, thereby demonstrating a pronounced neuroprotective effect against scopolamine‐induced Alzheimer's disease. These findings suggest that hordenine may exert its therapeutic action by attenuating oxidative stress and neuroinflammation, thereby mitigating cognitive decline. Furthermore, hordenine appears to preserve the integrity of the brain's histological structure, offering potential as a candidate for reversing neurodegenerative processes associated with Alzheimer's disease.

